# Diagnostic accuracy of intrathecal fluorescein versus other radiological modalities in evaluating non-congenital skull base defects: a systematic review and meta-analysis

**DOI:** 10.1007/s00405-024-08603-2

**Published:** 2024-04-06

**Authors:** Salma S. AlSharhan, Hussain J. Aljubran, Danah F. Alrusayyis, Aishah A. AlGhuneem, Wasan F. AlMarzouq, Mohammed H. Al Bar, Abdulmalik S. AlSaied, Mona M. Ashoor, Abdulaziz S. ALEnazi, Amal A. Alghamdi

**Affiliations:** 1 Department of Otolaryngology-Head and Neck Surgery, Imam Abdulrahman Bin Faisal University, King Faisal Ibn Abd Al Aziz, Al Rakah Ash Shamaliyah, 34221 Dammam, Saudi Arabia; 2 College of Medicine, Imam Abdulrahman Bin Faisal University, Dammam, Saudi Arabia; 3 Department of Family and Community Medicine, College of Medicine, Imam Abdulrahman Bin Faisal University, Dammam, Saudi Arabia

**Keywords:** Cerebrospinal fluid rhinorrhea, Cranial base, Intrathecal fluorescein, Intraoperative complications, Endoscopic surgery

## Abstract

**Purpose:**

The intraoperative detection of cerebrospinal fluid (CSF) leaks during endoscopic skull base surgery is critical to ensure watertight sealed defects. Intrathecal fluorescein (ITF) is a valuable adjunct to intraoperative investigation. Hence, our aim is to summarize the evidence of the efficacy of ITF as an accurate diagnostic modality and reconstruction guide for non-congenital skull base defects.

**Methods:**

Using the Cochrane Central, MEDLINE, and Embase databases, we identified studies involving the use of ITF in non-congenital CSF leaks which were published until November 2023. The STATA 18 software was used for meta-analysis.

**Results:**

Fourteen studies met the inclusion criteria, in which seven studies were included in the meta-analysis. ITF was used in 1898 (90.3%) of patients, with a detection rate of 88.1%. The overall detection rate of non-congenital CSF leaks among ITF concentrations of 5% and 10% had a statistically significant pooled effect size of 2.6 (95% CI = 2.25, 2.95), while when comparing the ITF to other alternative radiological tests, it was not statistically significant with a mean difference of 0.88 (95% CI = − 0.4, 2.16). Moreover, the pooled prevalence was statistically significant in regards of the complications associated with ITF with an effect size of 0.6 (95% CI = 0.39, 0.82), indicating that 60% of patients who underwent ITF would experience at least one of the measured complications.

**Conclusion:**

ITF is considered as an efficient tool in localizing skull base defects. However, there was no significant results when comparing the ITF to other alternative radiological tests. Accordingly, if the ITF intervention is indicated, patients should be carefully selected based on their clinical need.

## Introduction

Various methods have been implemented to improve the detection of cerebrospinal fluid (CSF) leakage sites, including intrathecal fluorescein (ITF), first described in 1960 by Kirchner and Proud [1]. Although ITF is not commonly used, it can help identify, localize, and repair CSF skull base defects [2–4]. The presence of green/yellow fluid delineates the CSF leak location 30 min after fluorescein administration. The sensitivity of ITF ranges from 46 to 100%, and the surgical success rate can be as high as 96% [5–15]. This wide range is attributed to various factors, including the fluorescein dose and concentration. For example, at a dose of 10 mg, the sensitivity of ITF is 73.8% [16], while at a dose of 25 mg, the sensitivity and specificity can reach 92.9% and 100%, respectively [17]. Despite being a desirable option when determining the extent of reconstruction, there is a lack of comprehensive systematic reviews investigating the efficacy and safety of non-FDA-approved ITF in detecting CSF leaks in non-congenital defects. Therefore, this systematic review aimed to search the literature for evidence of the importance of ITF as an accurate diagnostic modality and reconstruction guide for non-congenital skull base defects. Furthermore, we aimed to analyze the factors contributing to ITF-related complications and provide evidence-based guidelines on safe dosages and techniques.

## Methods

### Literature search

This systematic review followed the Cochrane review methods and the Preferred Reporting Items for Systematic Reviews and Meta-Analyses (PRISMA) guidelines (PROSPERO CRD42021273630) [18]. A literature search of the Cochrane Central, Medline, and Embase databases was conducted systematically on February 2021 and updated on November 2023, to identify articles published from 1960 onward with the following search strategy: (“CSF” [All Fields]) OR (“Cerebrospinal fluid” [All Fields]) OR (“Cerebrospinal fluid” [MeSH Terms]) AND (“Fluorescein”[All Fields]) OR (“Fluorescein” [MeSH Terms]) AND (“Intrathecal” [All Fields]) OR (“injections, spinal” [MeSH Terms]). The reference lists of the included articles were manually appraised to identify additional publications not found in the primary literature search.

### Study selection

This systematic review included English and non-English studies assessing the safety and efficacy of ITF in CSF leak localization and as an intraoperative guide for skull base reconstruction. Studies met the inclusion criteria if they included skull base defects of traumatic or spontaneous etiologies repaired using endoscopic procedures. However, studies comprising congenital malformations or those who did not use ITF in diagnosing CSF leaks in any patient in their sample or those who used Extracranial approach were excluded. Nonhuman studies, review articles, narrative reviews, meta-analyses, systematic reviews, reports involving fewer than five cases, animal studies, cadaver studies, and editorials were also excluded. To meet the scope of this systematic review, three independent authors (D.R., H.J., and A.G.) screened the studies in three steps before final inclusion. When disagreements occurred, the three authors discussed the article until a consensus was reached or the decision was made based on the majority.

### Data extraction

A single investigator retrieved data from a standardized database for each full-text article. General information included the first author’s name, publication year, country, study design, population size, median age, sex, and CSF leak etiology. Characteristic analyses of ITF included the preprocedural setting, fluorescein dosage and technique of administration, use of lumbar drains (LD), and detection rate. The surgical approach for reconstruction, follow-up period, ITF-related complications and CSF leak recurrence, were documented and linked to adverse outcomes. Furthermore, investigations used as an adjunct to ITF and their diagnostic rates were documented.

### Quality of the studies

The quality of the included studies was assessed according to the Enhancing the Quality and Transparency of Health Research (EQUATOR) reporting statement of observational studies (STROBE) with a maximum score of 22.

### Statistical analysis

In the tables, patient demographics, ITF characteristics, and outcome statistics were described using standard descriptive statistics. All fluorescein-related outcomes were reported as frequencies and percentages. The median age of the study population was reported, and the range was considered whenever the median was not mentioned. Additionally, meta-analysis was done using STATA 18 software. The models were done using random effect models to estimate the pooled log odds ratio and results were presented in forest plot. In addition, heterogeneity was tested using Cochran’s Q test and Higgins’s I^2^. Publication bias was investigated using funnel plot.

## Results

**Fig. 1:**
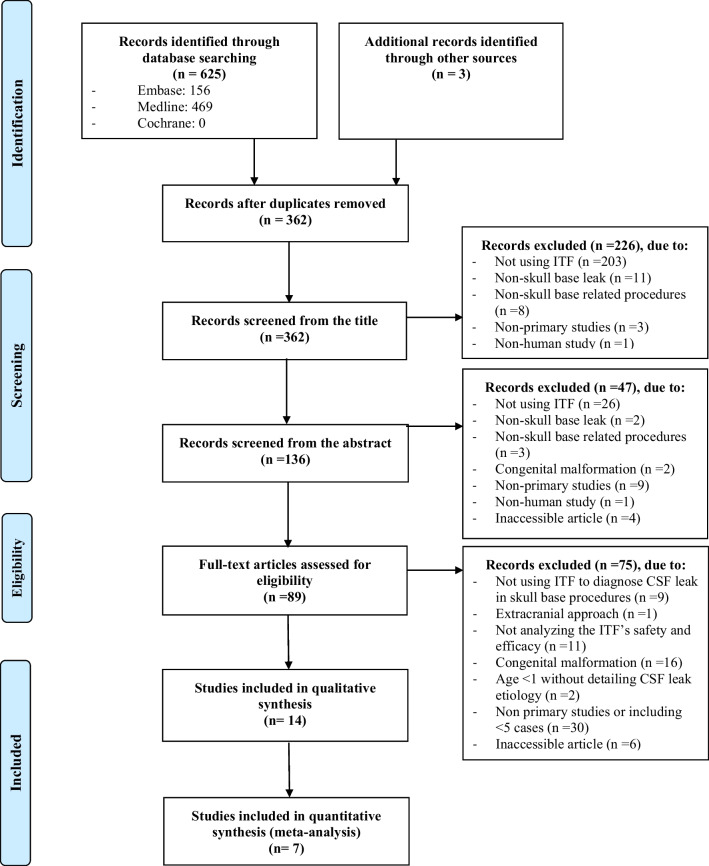
PRISMA flow diagram of systematic review process

The search identified 362 unique articles. Eighty-nine studies were selected for full-text review after screening the titles and abstracts for eligibility based on the exclusion criteria. Fourteen original studies (2101 patients) were finally included after reviewing the full text; the remaining studies were excluded for reasons stated in the PRISMA diagram (shown in Fig. 1).

### Study and population characteristics

The studies were either retrospective or prospective observations published between 1960 and 2023 in the United States [3, 17, 19, 20], Brazil [6, 21, 22], Germany [23, 24], India [25, 26], Italy [27], Iran [28], and Pakistan [29]. The patients were aged 1–86 years; however, the patients’ ages were unspecified in four studies. Nine studies focused primarily on ITF use [3, 6, 17, 19, 20, 22, 24, 28, 29], whereas the rest reported their surgical practice in CSF rhinorrhea management [21, 23, 25–27]. Of the 2101 individuals, 1898 (90.3%) had ITF as part of their management strategy for CSF leak repair. The CSF leak etiology was purely iatrogenic in Jakimovski et al. due to endoscopic pituitary surgery [19], idiopathic in the Englhard et al. cohort [23], due to skull base lesions in the Raza et al. cohort [17], and mixed traumatic and non-traumatic in the 11 remaining studies. Only six studies reported the efficacy of other diagnostic modalities, such as endoscopic inspection, beta 2-transferrin and beta trace protein, and radiological imaging [3, 21–23, 25, 27]. The characteristics of each study are presented in Table 1.

**Table 1:** Baseline characteristics of included articles

#	Author (Year)	Country	Study design	Sample size: (Total; ITF cases)	Age (median in years)*	Gender (n,%)	Etiology of CSF leak	Surgical approach	Detection rate
1	Wolf, et al. (1997) [24]	Germany	Retrospective study	(925; 925)	37.7	–	Traffic accident 51 patients (43%), tumor 9 patients (7%), sucide 8 patients (7%), spontaneous 7 patients (6%), other traumas 44 patients (37%)**	–	97 /119** (81.5%)
2	Guimarães, et al. (2002) [6]	Brazil	Retrospective study	(23; 23)	4–57 (range)	13 Males (56.5%) 10 Females (43.5%)	Traumatic in 15 patients (65%); nontraumatic in 8 patients (35%)	–	20/23 (86%)
3	Landeiro, et al. (2004) [21]	Brazil	Retrospective study	(10; 4)	18–67 (range)	7 males (70%) 3 females (30%)	Traumatic in 7 patients; traumatic iatrogenic in 2 patients; idiopathic in 1 patient	Endonasal endoscopic approach	4/4 (100%)
4	Demarco, et al. (2007) [22]	Brazil	Prospective study	(18; 18)	35	10 Males (55.6%) 8 Females (44.4%)	Idiopathic in 10 patients; traumatic in 7 patients; iatrogenic in 1 patient	Endonasal endoscopic approach	18/18 (100%)
5	Bhalodiya and Joseph. (2009) [25]	India	Retrospective and prospective study	(20; 20)	–	12 Males (60%) 8 Females (40%)	Traumatic in 14 patients (70%); non-traumatic in 6 patients (30%)	Endonasal endoscopic approach	20/20 (100%)
6	Javadi, et al. (2013) [28]	Iran	Case series	(20; 20)	33.7	15 Males (75%) 5 Females (25%)	Traumatic in 11 patients; iatrogenic in 6 patients; idiopathic in 3 patients	Endonasal endoscopic approach	18/20 (90%)
7	Jakimovski, et al. (2014) [19]	USA	Prospective cohort	(203; 203)	–	104 Males (51%) 99 Females (49%)	Traumatic iatrogenic (endoscopic pituitary surgery)	Endonasal endoscopic approach	189/203 (93.1%)
8	Emanuelli, et al. (2015) [27]	Italy	Retrospective study	(20; 8)	71	9 Males (45%) 11 Females (55%)	Traumatic/traumatic iatrogenic in 10 patients; idiopathic in 10 patients	Endonasal endoscopic approach	8/8 (100%)
9	Raza, et al. (2016) [17]	USA	Prospective cohort	(419; 419)	–	–	Skull base lesion	Endonasal endoscopic approach	400/419 (95.5%)
10	Englhard, et al. (2018) [23]	Germany	Retrospective study	(24; 24)	–	9 Males (37.5%) 15 Females (62.5%)	Idiopathic. Empty sella was detected in 1 patient (4.2%); elevated ICP detected in 4 patients (16.7%); normal pressure hydrocephalus detected in 1 patient (4.2%)	Endonasal endoscopic approach	21/24 (88%)
11	Keshri, et al. (2019) [26]	India	Retrospective study	(43; 15)	37.50	11 Males (25.6%) 32 Females (74.4%)	Idiopathic	Endonasal endoscopic approach	15/15 (100%)
12	Flynn, et al. (2020) [3]	USA	Retrospective study	(102; 56)	49.5	27 Males (26.5%) 75 Females (73.5%)	Idiopathic in 38 (39.6); iatrogenic in 27 patients (28.1); skull base tumor in 13 patients (13.5); traumatic in 9 patients (9.4); unknown in 9 patients (9.4)	Endonasal endoscopic approach	36/56 (64.28%) (multiple defects 9/9, single defects 27/47)
13	Mahmood, et al. (2021) [29]	Pakistan	Case series	(62; 62)	42.85	23 Males (37.1%) 39 Females (62.9%)	Traumatic and idiopathic	Endonasal endoscopic approach	49/62 (79%)
14	Radabaugh, et al. (2022) [20]	USA	Retrospective study	(212; 101)	48.8	27 Males (27%) 74 Females (73%)	Traumatic in 48 patients, idiopathic intracranial hypertension in 102 patients, tumor in 62 patients	Endonasal endoscopic approach	67/101 (66.3%)

*In cases where median is not mentioned, the age range in years is considered. **Data only mentioned for patients who underwent surgical procedure from 1978 to 1995

### ITF preparation and diagnostic efficacy

Table 2 details ITF preparations and their association with positive outcomes. In most studies, ITF was injected intraoperatively after induction of anesthesia and before endoscopic exposure. Guimarães et al. and Bhalodiya et al. reported that lumbar puncture (LP) was performed before anesthesia, except in children and anxious elderly patients [6, 25]. In two studies involving 622 participants, patients were premedicated with 10 mg of intravenous dexamethasone (unless diagnosed with Cushing’s disease) and 50 mg of diphenhydramine [17, 19]. Other authors administered perioperative antibiotics to 77 patients with ITF [22, 23, 25, 26]. Although ITF preparations were notably heterogeneous among the studies, 962 of 1092 (88.1%) patients were diagnosed. CSF was the sole dilution medium used in eight studies with 791 participants. Regarding the fluorescein formula, 10 mL of CSF was mixed with 0.1–0.25 mL of 10% fluorescein in 779 participants, and it helped to diagnose 692 (88.8%) [3, 17, 19, 20]. A dose of 0.05 mL/kg to a maximum of 1.0 mL of 5% fluorescein helped diagnose 51 of 56 subjects (91.1%) [21, 23, 27, 28]. Mixing 0.1 mL of 10% fluorescein had a detection rate of up to 66.3%, compared to the 100% noted with 0.2–0.7 mL of 5% fluorescein [3, 20, 25, 26]. A German study reported that injecting 0.5–1.0 mL of 5% sodium fluorescein via LP without mixing helped diagnose 97 of 119 operated cases (81.5%) [24]. Mahmood et al. Reported the highest fluorescein formula as they used CSF of 0.1 mL mixed with 25% fluorescein which helped in diagnosing 49 patients out of 62 patients, with a detection rate of 79% [29]. In all studies, successful reconstruction of the skull base defect was achieved in 80–100% of the cases after the first intervention and 100% after the revision surgery with persistent leaks.

**Table 2:** The Effect of ITF-Related Factors on the Intraoperative Outcomes and Recurrence

Author	Information related to the if exposure						Intraoperative outcomes			Post-operative surveillance	
	Time/venue of administration	ITF premedication	Dose	Fluorescein %	LD vs LP	Patient positioning	Detection rate	Diagnostic accuracy	Successful reconstruction	Follow-up	Recurrence
Mahmood, et al. (2021) [29]	Intraoperatively following the induction of anesthesia	–	10 mL of CSF mixed with 0.1 mL of fluorescein	25%	LD	Trendelenburg position	49/62 (79%)	–	–	2 weeks	0
Jakimovski, et al. (2014) [19]	Intraoperatively following the induction of anesthesia	IV 10 mg of dexamethasone and 50 mg of diphenhydramine	10 mL of CSF mixed with 0.25 mL of fluorescein	10%	LD were placed preoperatively in only 1 patient with a tumor < 2 cm in diameter and in 43 cases (35%) of tumors > 2 cm	–	189/203 (93.1%)	–	Postoperative CSF leak occurred in only 6 (3%) of patients, all of them were on LP	–	2 patients
Raza, et al. (2016) [17]	Intraoperatively as a first step before endonasal approach exposure	IV dexamethasone (10 mg) and IV diphenhydramine (50 mg). Patients with Cushing’s disease patients were not pretreated with dexamethasone	10 mL of CSF mixed with 0.25 mL of fluorescein	10%	LP (45.8%) LD (54.2%)	–	400/419 (95.5%)	Sensitivity (92.9%) Specificity (100%) NPV (88.8%) PPV (100%) True positive (59.7%) False positive (0%) False-negative (4.5%) True-negative (35.8%)	–	Mean: 34.6 months (range: 2–106)	7 patients (in the first 30 days post-op)
Flynn, et al. (2020) [3]	Intraoperative	–	10 mL of CSF mixed with 0.1 mL of fluorescein	10%	LD	–	36/56 (64.28%) (multiple defects 9/9, single defects 27/47)	–	4 persistent CSF leaks after surgery. 2 were managed conservatively with LD and 2 were managed with revision surgery	–	–
Radabaugh, et al. (2022) [20]	Intraoperatively. following the induction of anesthesia	–	0.1 mL of a fluorescein solution diluted in either 10 mL of sterile 0.9% normal saline solution or 10 mL of CSF	10%	LP (5%) LD (95%)	–	67/101 (66.3%)	Sensitivity (66.3%) Specificity (100%) False-negative (33.6%)	–	330 days	10 patients
Bhalodiya and Joseph. (2009) [25]	Before anesthesia, except in children and more anxious elderly patients	Aetazolamide and laxatives	10 mL of CSF mixed with 0.2–0.7 mL of fluorescein	5%	LD	30 degrees in children and very anxious elderly patients after general anesthesia	20/20 (100%)	100%	85%	Mean is 2 years	3 patients
Keshri, et al. (2019) [26]	Intraoperative	Oral cefpodoxime 200 mg, 12th hourly for 10 days and oral acetazolamide 250 mg, 6th hourly with syrup potassium chloride 5 to 10 mL daily	0.2 mL of fluorescein for < 60 kg and 0.25 mL for > 60 kg body weight	5%	LP or LD	Trendelenberg position	15/15 (100%)	–	95.3% (41 out of 43 patients)	18 months (range: 6–36 months)	2 patients (4 and 6 months post-op)
Landerio, et al. (2004) [21]	Intraoperatively following the induction of anesthesia	–	10 mL of CSF fluid mixed with 0.1 mL fluorescein	5%	LP	Head of patient placed slightly below the heart level	4/4 (100%)	–	100%	–	1 patient (14 months post-op)
Javadi, et al. (2013) [28]	Intraoperatively, following the induction of anesthesia	–	10 mL of CSF withdrawn and mixed with 1 mL of flourscein	5%	LP	Lateral decubitus position	18/20 (90%)	–	80%	6 months	4 patients
Emanuelli, et al. (2015) [27]	Intraoperatively following the induction of anesthesia	–	9 mL of CSF mixed with fluorescein (at the dose of 0.05 mL/10 kg)	5%	LP	Trendelenburg position	8/8 (100%)	–	100%	6 to 24 months	0
Englhard, et al. (2018) [23]	–	All patients given antibiotics peri- and post-operatively for at least 3 days	0.1 mL / 10 kg of body weight, maximum 1 mL	5%	LP (33.3%) LD (66.7%)	Tilted into a head-down position	21/24 (88%)	–	80% of primary and 100% of secondary repair were successful	12 months	5 patients (day 2, 3, 5, 14 and 192 post-op)
Guimarães, et al. (2002) [6]	Before anesthesia, except in children and more anxious elderly patients	–	0.5 cm^3^ of fluorescein diluted in 10cm^3^ of distilled water, solution with density of 1001	5%	LD	Elevated head and trunk	20/23 (86%)	–	–	–	–
Demarco, et al. (2007) [22]	Approximately 5 min before surgery	IV ceftiaxone (2g)	0.1 mL/10 kg of body weight of sodium fluorescein solution (maximum dose is 1 mL) diluted in 10 mL of distilled water	5%	LP	Trendelenburg position	18/18 (100%)	94.4%	88% in the 1st intervention 100% in the 2nd intervention	23 months	2 patients
Wolf, et al. (1997) [24]	–	–	0.05–0.1 mL sodium fluorescein per 10 kg per weight, but never a larger total amount than 1 mL	5%	Sub-occipital puncture 1971–1990 then LP was used up to 1995	Prone head-down position	97 /119* (81.5%)	Correct localization (69%) Misinterpretation (13%) False negative (1–7%)	–	–	–

ITF: intrathecal fluorescein. CSF: cerebrospinal fluid. LP: lumbar puncture. LD: Lumbar drain. VPS: ventriculoperitoneal shunt. *Detection rate is only mentioned for patients who underwent surgical procedure from 1978 to 1995

### Follow-up and recurrence

The follow-up period ranged from 2 weeks to 106 months. CSF leak recurrence has been discussed in 11 of 14 studies involving 894 participants [17, 20–23, 25–29]. Only 36 patients (4%) had evidence of recurrence and were treated with revision surgery or conservatively through LD. Radabaugh et al. reported the highest recurrence rate of CSF leakage (9.9% of patients) [20]. Table 2 summarizes the recurrence rates and follow-up periods of the included studies.

### ITF-related complications

Table 3 briefly summarizes the studies that assessed perioperative ITF complications. The reported complication rates of ITF injections ranged from 0 to 20%. Overall, 45 of 1,898 patients developed complications (2.4%). Seven studies reported the presence of ITF-related complications during the follow-up period [3, 19, 20, 22, 24–26]. Most of these complications were transient, except for one study that included a patient with permanent diplopia and 12 patients with endocrinopathies [19]. Neurological complications (e.g., seizure, meningitis, syncopal episodes, headaches, and anosmia) were the most common perioperative complications which reported in 22 patients which accounts for 48.9% of the overall complications [3, 19, 20, 22, 24, 26]. Javadi et al. reported late complications after ITF, including meningitis, pneumocephalus, and pseudoaneurysm [28]. A total of 779 subjects were injected with 0.1–0.25 mL of 10% fluorescein mixed with 9–10 mL of CSF, which was associated with complications in 31 cases (4.0%) that were primarily related to endocrinopathies [3, 17, 19, 20]. Five percent fluorescein at 0.05 mL/kg to a maximum of 1.0 mL was used in 56 participants, with complications in four cases (7.1%), all classified as late complications [21, 23, 27, 28]. In two other studies with 41 participants, 0.5–1 mL of 5% sodium fluorescein was diluted in 10 mL of distilled water and was associated with headache in only one patient (2.4%) [6, 22]. According to Wolf et al., injecting 0.5–1.0 mL of 5% sodium fluorescein through the LP without mixing resulted in seizures in three of 119 patients (2.5%) [24].

**Table 3:** Potential patient- and fluorescein-related contributing factors to the development ITF complications

Author	Patients Characteristics		IF administration protocol							Follow-up	Complications			
	Median age (years)	Comorbidities	Time/venue of administration	ITF premedication	Dose	Fluorescein %	LD vs LP	Technique of administration	Patient positioning		Rate	Peri-operative	Late	Nature
Jakimovski, et al. (2014) [19]	–	–	Intraoperatively following the induction of anesthesia and before endonasal approach exposure	IV 10 mg of dexamethasone and 50 mg of diphenhydramine	10 mL of CSF mixed with 0.25 mL of fluorescein	10%	LD were placed preoperatively in only 1 patient with a tumor < 2 cm in diameter and in 43 cases (35%) of tumors > 2 cm	Injected slowly over several minutes	–	–	12.3%	Post-operative endocrinolopathies (n = 12; 4 adrenal insufficiency, 8 diabetes insipidus) Postoperative perisellar hematomas (n = 3) Systemic complication (n = 4; 3 pulmonary embolism, 1 dehydration) Bacterial meningitis associated with postoperative CSF leaks (n = 2) Diplopia (n = 2; both had tumor invading the cavernous sinus) Hypotension (n = 1) Unexplained syncopal episodes (n = 1)		Transient and permanent
Flynn, et al. (2020) [3]	49.5	–	Intraoperative	–	10 mL of CSF mixed with 0.1 mL of fluorescein	10%	LD	injected over a 10-min period. The LD remained in place for varying amounts of time postoperatively	–	–	10.7%	Spinal headache (n = 3) Seizure (n = 3)	–	–
Bhalodiya and Joseph. (2009) [25]	–	Allergic rhinitis (n = 3) Hypertension (n = 3)	Before anesthesia, except in children and more anxious elderly patients	Aetazolamide and laxatives	10 mL of CSF mixed with 0.2–0.7 mL of fluorescein	5%	LD	Injected at L3/L4 or L4/L5	30 degrees in children and very anxious elderly patients after general anesthesia	Mean = 24 months	20%	Anosmia (n = 4)	–	Transient
Keshri, et al. (2019) [26]	37.5	–	Intraoperative	Oral cefpodoxime 200 mg, 12th hourly for 10 days and oral acetazolamide 250 mg, 6th hourly with syrup potassium chloride 5 to 10 mL daily	10 mL of normal saline mixed with 0.2 mL of fluorescein for < 60 kg and 0.25 mL for > 60 kg body weight	5%	LP or LD	injected into the subarachnoid space over 10 min	Trendelenberg position	Mean = 18 months (range: 6–36 months)	13.3%	Meningitis (n = 2)	–	Transient
Javadi, et al. (2013) [28]	33.7	–	Intraoperatively, following the induction of anesthesia	–	10 mL of CSF withdrawn and mixed with 1 mL of flourscein	5%	LP	Injected slowly over several minutes	Lateral decubitus position	6 months	20%	–	Meningitis (n = 2) pnemocephalus (n = 1) pseudoaneurysm (n = 1)	Transient
Demarco, et al. (2007) [22]	35	–	Approximately 5 min before surgery	IV ceftiaxone (2 g)	0.1 mL/10 kg of body weight of sodium fluorescein solution (maximum dose is 1 mL) diluted in 10 mL of distilled water	5%	LP	Slowly by over a period of 5 min	Trendelenburg position	23 months	5.5%	Headache (n = 1)	–	Transient
Wolf, et al. (1997) [24]	37.7	-	–	–	0.05–0.1 mL sodium fluorescein per 10 kg per weight, but never a larger total amount than 1 mL	5%	Sub-occipital puncture 1971–1990 then LP was used up to 1995	Slowly injected intrathecally	Prone head-down position	–	2.5%	Grand-mal seizure with sub-occipital injection of ITF (n = 3)		Permanent (n = 1) Transient (n = 2)

Only studies that reported complications after fluorescein injection were detailed. ITF: intrathecal fluorescein. *CSF* cerebrospinal fluid, *LP* lumbar puncture, *LD* lumbar drain, *VPS* ventriculoperitoneal shunt. Age range is considered whenever the median is not reported

### Adjunct diagnostic modalities

ITF was not used alone and was considered an adjunct to localize leaks as the authors performed diagnostic imaging on all participants. However, only seven studies reported the diagnostic rates of radiological tests (Table 4). The collective diagnostic rate of the 237 patients with alternative investigations was 75.5%, compared to 84.1% for ITF cases.

**Table 4:** Comparison between the detection rates of ITF and alternative tests utilized in studies’ cohorts

Author and year	Utilization of diagnostic studies		Detection rate	
	Dose and fluorescein %	Name of the alternative test	Detection rate of ITF	Detection rate of alternative tests
Bhalodiya and Joseph (2009) [25]	0.2–0.7 mL of 5% sodium fluorescein solution mixed with 10 mL of CSF or distilled water	CT/MR cisternogram	20/20 (100%)	17/20 (85%)
Keshri, et al. (2019) [26]	0.2–0.25 mL of 5% sodium fluorescein solution mixed with 10 mL of normal saline	CT/MR cisternogram	15/15 (100%)	28/43 (65.1%)
Landerio, et al. (2004) [21]	10 mL of CSF fluid adding 5% sodium fluorescein in a rate of 0.1 mL/kg with a limit rate of 1 mL of the solution	CT scan	4/4 (100%)	6/10 (60%)
Flynn, et al. (2020) [3]	10 mL of CSF to be mixed with 0.1 mL of 10% fluorescein	HRCT	36/56 (64.28%)	86/102 (84.3%)
Emanuelli, et al. (2015) [27]	9 mL of CSF were withdrawn, mixed with 5% sodium fluorescein solution (at the dose of 0.05 mL/10 kg) and slowly injected intrathecally. The maximal dose never exceeded 50 mg of sodium fluorescein	HRCT	8/8 (100%)	12/20 (60%)
Englhard, et al. (2018) [23]	0.1 mL / 10 kg of body weight, maximum 1.0 mL of 5% fluorescein	HRCT	21/24 (87.5%)	13/24 (54.2%)
Demarco, et al. (2007) [22]	0.1 mL/10 kg of body weight of 5% sodium fluorescein solution (maximum dose is 1 mL) diluted in 10 mL of distilled water	HRCT	18/18 (100%)	17/18 (94.4%)

Only studies that clearly stated the detection rate of an alternative tests were included in this table. *ITF* intrathecal fluorescein, *CSF* cerebrospinal fluid, *HRCT* high-resolution computed tomography, *CT* computed tomography, *MR* magnetic resonance, *MRI* magnetic resonance imaging

### Assessment of bias

The quality assessment of each study is presented in Supplement 1. The mean score of the included studies using the STROBE statement was 15.9. The maximum score obtained was 21 of 22. Almost all studies properly elaborated on the scientific background, study design and settings, and descriptive outcome data. All studies provided a cautious overall interpretation of the results, considering the objectives and results of similar studies. The weakest aspect of the studies was that the findings were reported as descriptive statistics. Accordingly, confounder-adjusted estimates and their precision were not reported except in three studies [19–21]. Only three studies clearly stated the potential sources of bias [3, 17, 19].

### Meta-analysis results

**Fig. 2:**
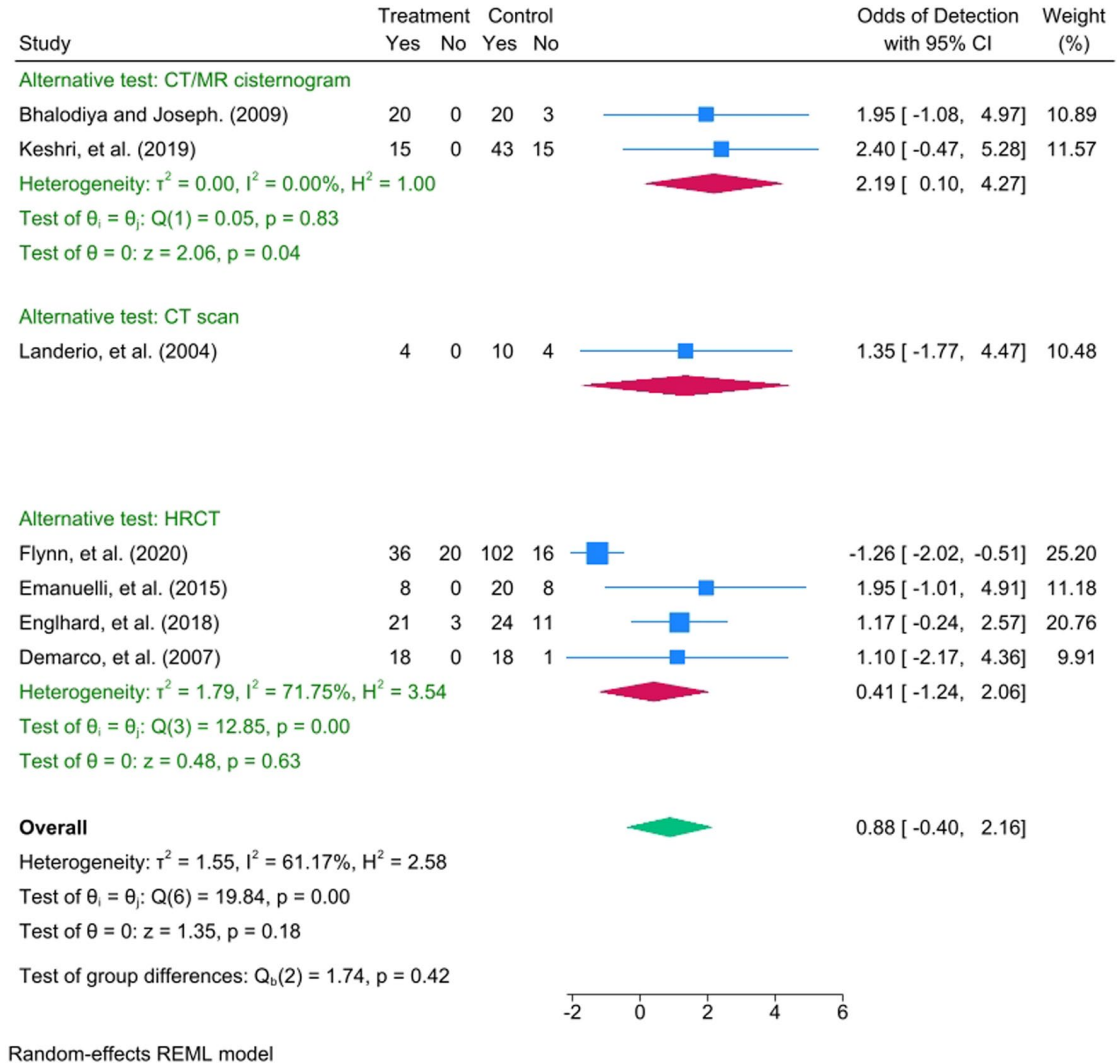
Forest plot which shows the mean difference between ITF and alternative radiological tests in detecting CSF leak

**Fig. 3:**
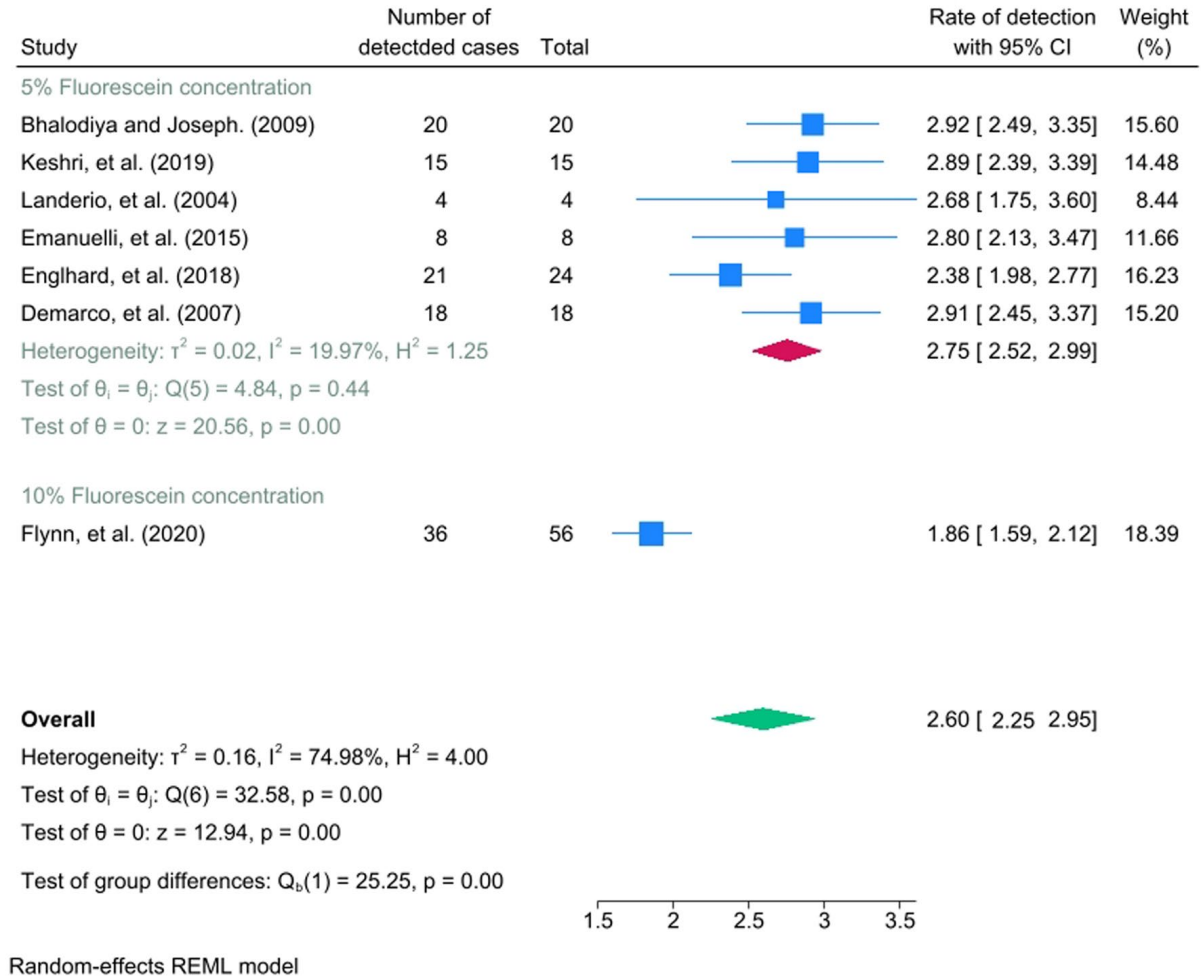
Forest plot which shows the detection rate of CSF leak in 5% and 10% fluorescein concentrations

**Fig. 4:**
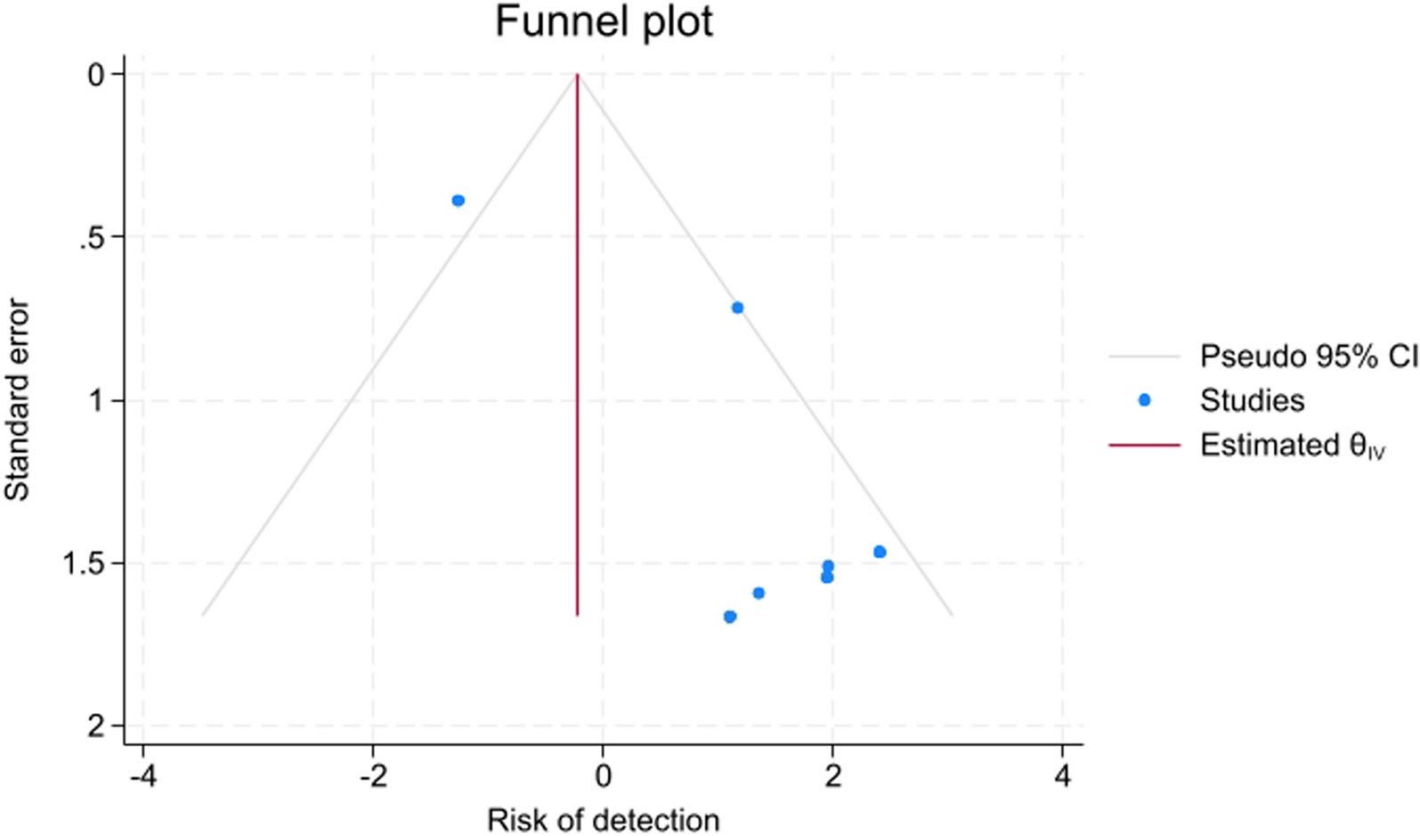
Funnel plot which investigated the presence of publication bias

**Fig. 5:**
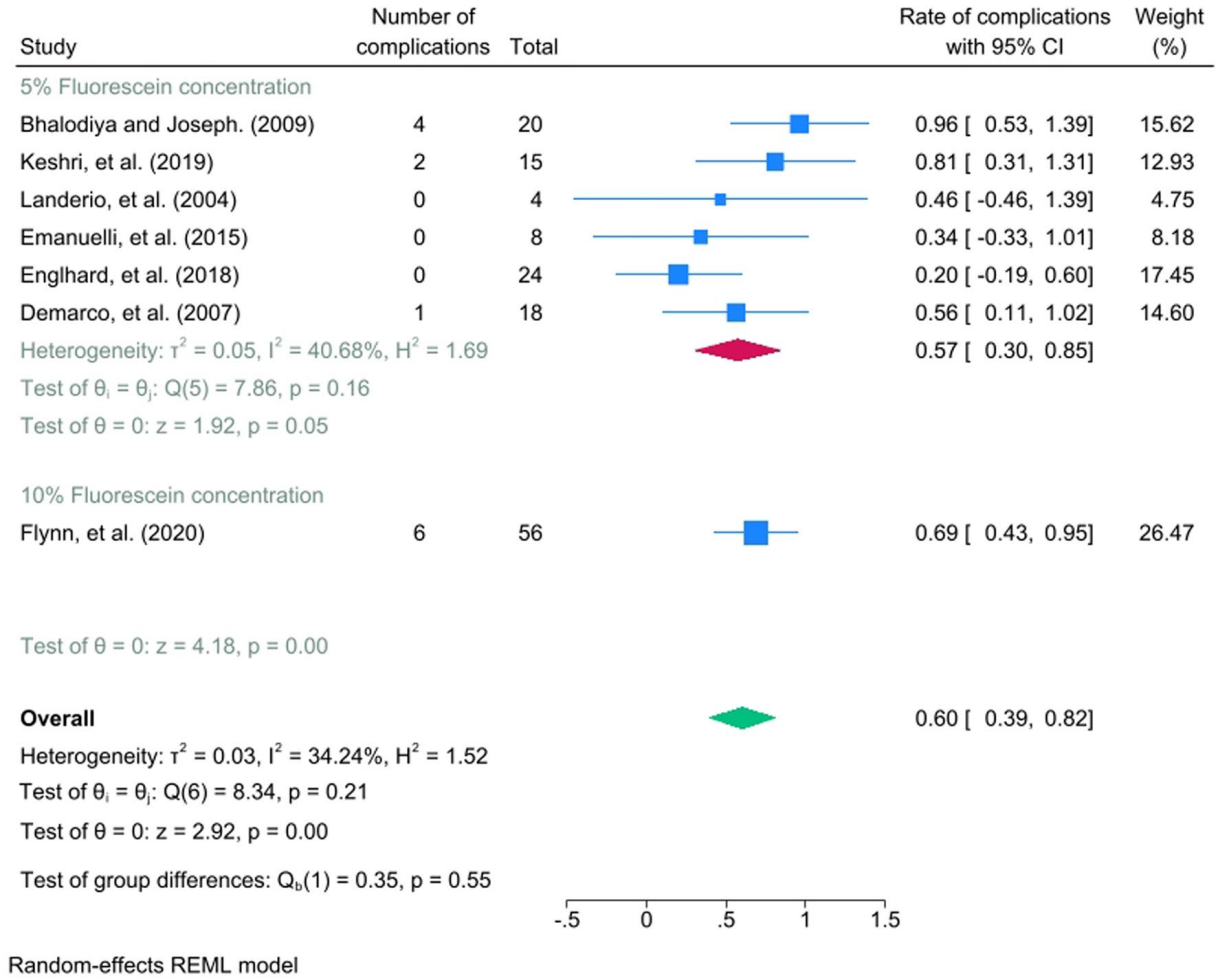
Forest plot which shows the complication rate of CSF leak in 5% and 10% fluorescein concentrations

Seven studies have been involved in the meta-analysis out of the fourteen studies. The pooled effect size of the odds ratio for identifying non-congenital CSF leak when comparing the ITF to other alternative radiological tests (such as CT/MRI cisternogram, High-resolution Computed Tomography, and CT scan) was not statistically significant, showing a mean difference of 0.88 (95% CI = − 0.4, 2.16) with a heterogenicity of I^2^ = 61.17%, as seen in Fig. 2. The detection rate of non-congenital CSF leaks among ITF concentrations of 5% and 10% had a statistically significant pooled effect size of 2.6 (95% CI = 2.25, 2.95) with a heterogenicity reaching I^2^ = 74.98%, as seen in Fig. 3. The funnel plot in Fig. 4 illustrates the spread of study estimates in relation to the precision; however, due to the limited number of involved studies, the effectiveness of the funnel plot might be impacted. The skewed distribution of studies within the 95% CI indicates the potential for publication biases favoring the reporting of the higher ITF detection rate. Moreover, the pooled prevalence of the complications among the ITF patients was 0.6 (95% CI = 0.39, 0.82) with a heterogeneity reaching I^2^ = 34.24%, indicating that 60% of patients who underwent ITF would experience at least one of the measured complications, as seen in Fig. 5.

## Discussion

### ITF vs. radiological investigations: is ITF worth the risk?

Based on aggregated data from the studies included in this systematic review, all patients underwent radiological evaluations to diagnose CSF leakage, regardless of ITF use. High-resolution Computed Tomography was performed on all patients and was helpful in most cases, whereas some studies utilized magnetic resonance imaging (MRI) and MR/CT cisternography [30–38]. However, many skull base surgeons are undecided regarding whether ITF is worth the risk, considering its complications, compared to other diagnostic imaging modalities.

In a retrospective study by Flynn et al., 102 patients underwent CSF leak repair, and successful CSF leak identification was similar between preoperative CT and ITF injection when a single leak was present [3]. However, ITF had superior diagnostic ability than CT (100% vs. 81%) for multiple CSF leak sites [3]. Woodworth et al. found ITF useful in spontaneous CSF leaks, as it was superior for detecting multiple skull base defects in patients with benign intracranial hypertension [39]. This suggests the diagnostic value of ITF, irrespective of CT findings, as the presence of multiple leak sites is not always known preoperatively.

### Factors affecting the efficacy of ITF

The etiology of CSF leaks can affect ITF accuracy during localization, which may be due to intrinsic differences in the patho-etiological mechanisms of the leak. For example, in idiopathic intracranial hypertension, there is a dysfunction in the CSF absorptive mechanism of the arachnoid granulations or extracranial lymphatics [40]. In patients with trauma, CSF depletion and subsequent intracranial hypotension can limit the complete dissemination and visualization of fluorescein at the skull base. Another potential explanation for traumatic leaks is the formation of leptomeningeal fibrosis due to inflammatory reactions to blood products when there is hemorrhage in the subarachnoid space. This could restrict the flow of fluorescein-stained CSF into the defect [20, 40, 41]. The ITF process may also be affected by insufficient time from intrathecal injection to endoscopic intervention or uneven fluorescein distribution within the subarachnoid space [17].

In a series by Radabaugh et al., ITF had a false-negative rate of 33.6% and was insufficient when used alone to exclude CSF leaks [20]. Raza et al. reported a much lower false-negative rate (4.5%), but they used a higher fluorescein dosage (0.25 vs. 0.1 mL of 10% fluorescein) and focused mainly on skull base lesions as an etiology [17]. If the defect cannot be localized ITF, even after head-down positioning and positive pressure ventilation maneuvers, Englhard et al. advise terminating the procedure and running additional diagnostics (e.g., CT or MRI cisternography) to guarantee accurate localization [23].

### Relationship between ITF preparations and the development of complications

ITF utilization lacks Food and Drug Administration (FDA) approval and standardization, and there is no consensus among practitioners regarding its optimal utility. This poses potential risks to patients, and surgeons may face medicolegal problems [3]. Nevertheless, ITF is a second-line diagnostic tool for localizing skull base defects in operative settings and is potentially safe when used at recommended doses [3, 27, 42, 43]. Complications associated with ITF methods or doses have been reported [24, 25, 44–46]. High doses (500–1500 mg) are associated with various life-threatening complications, such as seizures, spinal cord injury, and even death. Although ITF has been reported to be safe at lower doses, it should be used cautiously, as a large meta-analysis by Felisati et al. reported that transient complications could occur even with ITF doses of < 50 mg [43]. Furthermore, this systematic review highlights varying preparations for ITF administration, such as hypodense fluorescein or diluting sodium fluorescein in distilled water instead of CSF. Such preparations are simpler and more effective in locating CSF fistulas, with no significant intra- or postoperative complications [22, 26, 27].

### Limitations

We acknowledge the limitations of our study. First, observational studies can have unmeasured confounders in addition to the recall bias noted in retrospective cohorts. Second, the clinical and statistical heterogeneity was substantial but expected since ITF is still off-label and there are no treatment guidelines for its preparations. However, to address heterogeneity, we carefully examined the included studies for mutual variables related to our aim and used unified metric units (whenever possible) for comparison. Third, 92 patients (four studies) did not undergo ITF, and it was challenging to distinguish whether recurrent CSF leaks occurred in this group or in patients whose leaks were initially localized by ITF. However, this issue was not encountered when other complications were examined. Fourth, we acknowledge the likelihood of publication bias, along with bias in the data acquisition and review processes. The use of predetermined, strictly defined inclusion and exclusion criteria, adoption of a data extraction strategy, and robust quality assessment conducted independently by the authors indicate that this systematic review included the highest quality of evidence in reporting the efficacy and safety of the current off-label administration of ITF in diagnosing CSF leaks. Finally, the scarcity of comparative studies between ITF and other diagnostic tests affected our ability to estimate the benefit-risk ratio. However, we acquired data on the detection rates of additional diagnostic tests whenever reported to demonstrate the value of adding ITF to the management plan.

## Conclusions

The effective rate of ITF for localizing CSF Leaks, reportedly having an overall detection rate of 46–100% and a surgical success rate up to 96%. Nevertheless, the surgical complication rates were reported to range from 0 to 25%. Although the need for ITF in the era of advanced radiological techniques is debated, as in our pooled results there was no significant difference in the detection rate between ITF and alternative radiological studies. Moreover, no randomized controlled trials were identified in the literature, and the heterogeneity in findings among studies means that this conclusion is supported by limited evidence. Accordingly, if the ITF intervention is indicated, Patients should be carefully selected based on their clinical need. Future studies should investigate the use of ITF combined with other diagnostic modalities to enhance its sensitivity and specificity for detecting CSF leaks.

## Data Availability

Data supporting this study are included within the article and supporting materials.
